# Assessment of patients with Covid-19 hospitalized in southern Santa Catarina

**DOI:** 10.1590/0037-8682-0579-2020

**Published:** 2020-11-25

**Authors:** Fabiana Schuelter-Trevisol, Leonan José Raimundo, Hadymilla Duarte Soccas, Ariana Francisco Antunes, Regina Longen Degering Mohr, Chaiana Esmeraldino Mendes Marcon, Daisson José Trevisol

**Affiliations:** 1 Universidade do Sul de Santa Catarina, Programa de Pós-Graduação em Ciências da Saúde, Tubarão, SC, Brasil.; 2 Associação Congregação de Santa Catarina, Centro de Pesquisas Clínicas, Hospital Nossa Senhora da Conceição, Tubarão, SC, Brasil.; 3 Hospital e Maternidade Socimed, Tubarão, SC, Brasil.

**Keywords:** Coronavirus infections, Hospitalization, Severe acute respiratory syndrome

## Abstract

**INTRODUCTION:**

Coronavirus disease 2019 (COVID-19), a potentially fatal disease, is caused by the severe acute respiratory syndrome coronavirus-2 (SARS-CoV-2). The number of cases has increased rapidly, but information on the clinical characteristics remains limited.

**METHODS:**

Cohort study. We collected and analyzed epidemiological, demographic, and clinical data of critically and noncritically ill patients and compared the outcomes.

**RESULTS:**

The mean age of hospitalized patients with COVID-19 was 54 years (standard deviation 16.9; 53.8% men), 29% required ICU admission, and 18.6% died.

**CONCLUSIONS:**

The main risk factors for ICU admission were age over 60 years, obesity, and preexisting chronic lung diseases.

Coronavirus disease (COVID-19) is caused by the severe acute respiratory syndrome coronavirus-2 (SARS-CoV-2) and is a potentially fatal disease of great global public health concern^1^. There have been approximately 13 million reported cases of COVID-19 and 573,572 deaths to date (07/15/2020). Up to mid-July, almost 2 million confirmed cases and approximately 75,000 deaths were recorded in Brazil^2^.

The clinical presentation and course of the disease are quite heterogeneous, ranging from a mild flu-like syndrome to a severe acute respiratory syndrome, a critical condition that requires intensive care and ventilatory support. It can also be associated with a systemic inflammatory response syndrome, hemodynamic instability, shock, heart failure, kidney failure, and death^3^ ^,^ ^4^. In moderate to severe cases, there is a need for hospitalization, sometimes in an intensive care unit (ICU). Some population groups are more vulnerable to infection than others, either because of immune system reduction or its inefficiency. In addition, some elderly people and those with comorbidities may have a large number of angiotensin-converting enzyme 2 (ACE-2) receptors, where SARS-CoV-2 binds through the spike protein to penetrate host cells and initiate its viral replication cycle^3^. Considering that this recent epidemic has a new etiological agent whose natural history has not yet been fully clarified, there is no effective treatment protocol, although recent evidence suggests that dexamethasone may reduce mortality in patients on mechanical ventilation^5^ and the use of prophylactic anticoagulants reduces the risk of thrombosis^6^. Despite supportive treatment for clinical manifestations in each patient, there is no consistency in the standardization of these protocols^3^.

Although some comorbidities and risk factors have already been identified as facilitating disease worsening and death, there are very few studies describing this issue in Brazilian populations. The aim of this study was to describe the hospitalized cases of COVID-19 in a microregion of the State of Santa Catarina by comparing critical cases and deaths in relation to demographic and clinical characteristics.

This prospective cohort study included hospitalized patients diagnosed with COVID-19 between March 16 and July 16, 2020, in the two hospitals that provide intensive care beds in the microregion of Tubarão, Santa Catarina, Brazil. We confirmed all cases with real-time polymerase chain reaction (RT-PCR) tests or serological tests associated with epidemiological criteria. The microregion is formed by 18 cities, with an estimated population of approximately 400,000 people, located in the southern region of the State of Santa Catarina, and is the headquarters of the Association of the Laguna Region Municipalities (AMUREL, in the Portuguese acronym). It was one of the first places to detect the start of community transmission of SARS-CoV-2 in mid-March 2020 and became the sixth highest in the number of cases in Brazil. Since the beginning of the epidemic, the two referral hospitals in the region have allocated infirmary and intensive care beds exclusively for the treatment of COVID-19. Information was collected on the date of onset of symptoms, date of hospitalization and hospital discharge, date of transfer to the ICU, age (in years), sex (male and female), comorbidities or underlying diseases (hypertension, diabetes, heart and lung diseases, dyslipidemia, obesity, smoking, vascular and kidney diseases, psychiatric disorders, or neurological diseases), and outcomes (discharge, death, or hospital transfer). 

The collected data were analyzed using SPSS v.21 (IBM, Armonk, NY, USA). Descriptive analysis was used for the quantitative and qualitative variables. Crude and adjusted binomial logistic regressions were performed to estimate the isolated effect of age, sex, and comorbidities in ICU admission and death. 

This study was approved by the Research Ethics Committee of the National Commission of Research Ethics (CONEP), following the ethical standards of Ethics Resolution 466 of 2012 through a report number 4,029,342 on May 15, 2020, in accordance with the principles in the Declaration of Helsinki.

In the first 120 days of the epidemic, 347 suspected cases of COVID-19 were hospitalized, of which 211 (60.8%) were confirmed. Figure 1 shows the distribution of hospitalized, suspected, and confirmed cases by epidemiological week.

**FIGURE 1:**
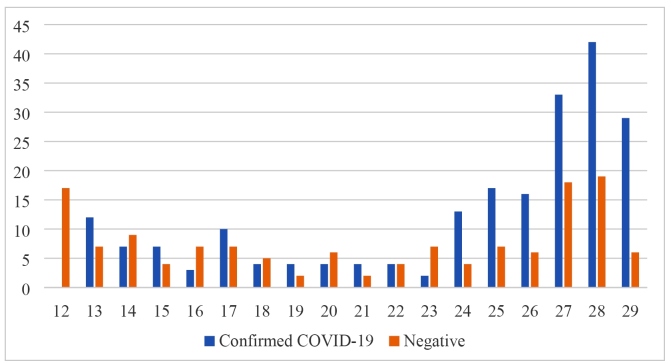
Distribution of hospitalized suspect or confirmed cases of COVID-19 by epidemiological week in Tubarão, Santa Catarina, Brazil.

Of the confirmed cases, the mean age was 54 years (standard deviation 16.9), ranging from 2 to 92 years. The median time spent seeking medical care was 7 days, considering the onset of symptoms. Table 1 shows the demographic and clinical profiles of cases hospitalized in the first 120 days of the epidemic in the region.

**TABLE 1:** Demographic and clinical characteristics of hospitalized patients with COVID-19 (n = 211).

Characteristics	n	%
Gender		
Male	113	53.6
Female	98	46.4
Age		
0-9	1	0.5
10-19	-	-
20-29	12	5.7
30-39	33	15.6
40-49	48	22.7
50-59	37	17.5
60-69	40	19.0
70-79	25	11.8
80-89	11	5.2
90-99	4	1.9
Comorbidities		
Arterial hypertension	67	31.9
Diabetes mellitus	37	17.6
Chronic heart diseases	27	12.9
Obesity	18	8.6
Neurologic/psychiatric diseases	17	8.1
Chronic lung diseases	15	7.1
Dyslipidemia	8	3.8
Smoking habits	6	2.9
Cancer	6	2.9
Chronic kidney diseases	5	2.4
Vascular diseases	3	1.4
ICU		
Yes	61	29.0
No	150	71.1
Pharmacological treatment		
Azithromycin	162	76.8
Hydroxychloroquine	15	7.1
Dexamethasone	102	48.3
Anticoagulants	188	89.1
Other corticosteroids	110	52.1
Disclosure		
Discharged	155	73.5
Death	41	19.4
Transfer to another hospital	4	1.9
Hospitalization	11	5.2
	Median	Min - Max
Time of hospitalization in days		
Infirmary	4	1-36
ICU	13	0-115

**ICU:** intensive care unit.

Table 2 shows the crude and adjusted analysis of the outcomes of admission to the ICU and death in relation to demographic and clinical variables.

**TABLE 2:** Crude and adjusted analysis of risk factors for admission to intensive care unit and death due to COVID-19 (n = 211).

	ICU				Death			
	OR crude	P-value	OR adjusted	P-value	OR crude	P-value	OR adjusted	P-value
	(CI 95%)		(CI 95%)		(CI 95%)		(CI 95%)	
Age ≥ 60 years old	3.14 (1.69-5.82)	<0.001	3.59 (1.67-7.69)	0.001	11.06 (4.70-26.00)	<0.001	10.67 (4.05-28.10)	<0.001
Male gender	1.50 (0.82-2.75)	0.188	-		1.48 (0.72-3.04)	0.281	-	
Comorbidities	3.29 (1.73-6.28)	<0.001	0.97 (0.35-2.69)	0.958	3.88 (1.73-8.71)	0.001	2.82 (0.90-8.90)	0.076
Arterial hypertension	2.65 (1.42-4.94)	0.002	1.10 (0.44-2.76)	0.840	2.62 (1.28-5.39)	0.009	0.59 (0.20-1.74)	0.341
Diabetes mellitus	1.63 (0.78-3.43)	0.197	-		2.09 (0.92-4.74)	0.077	-	
Chronic heart disease	2.61 (1.15-5.95)	0.022	2.09 (0.74-5.92)	0.165	2.86 (1.15-7.14)	0.024	0.98 (0.31-3.10)	0.974
Obesity	3.46 (1.29-9.24)	0.013	6.83 (1.93-24.25)	0.003	1.79 (0.59-5.42)	0.303	-	
Neurologic/psychiatric diseases	3.05 (1.12-8.33)	0.029	2.65 (0.78-8.96)	0.118	1.97 (0.64-6.03)	0.238	-	
Chronic lung diseases	4.13 (1.40-12.16)	0.010	4.04 (1.20-16.13)	0.025	3.01 (1.00-9.04)	0.050	2.54 (0.63-10.17)	0.188
Dyslipidemia	4.35 (1.01-18.79)	0.049	2.64 (0.48-14.68)	0.266	0.81 (0.09-7.10)	0.845	-	
Smoking habits	5.16 (0.92-28.94)	0.062	-		2.79 (0.45-17.32)	0.270	-	
Cancer	0.48 (0.06-4.20)	0.507	-		2.08 (0.37-11.80)	0.408	-	
Chronic kidney diseases	3.80 (0.62-23.34)	0.149	-		17.93 (1.95-165.49)	0.011	4.11 (0.40-42.00)	0.233
Vascular diseases	5.02 (0.45-56.38)	0.191	-		-	-	-	

**ICU:** intensive care unit; **OR:** Odds ratio; **CI:** confidence interval.

Patients who required admission to the intensive care unit were 15 times more likely to have unfavorable outcomes than those admitted to a ward (RR 15.7 [CI95% = 7.01-35.37], p <0.001). A longer hospital stay was also associated with the need for ICU admission (19 vs. 5 days; p <0.001), and patients who died also had a longer hospital stay compared to those with favorable outcomes (16 vs. 7 days; p <0.001).

There was a positive association between the use of hydroxychloroquine (p <0.001), anticoagulants (p = 0.006), dexamethasone (p = 0.022), and other corticosteroids (p <0.001), and ICU hospitalization. The use of hydroxychloroquine (p <0.001) and other corticosteroids (p <0.001) also showed a positive association in cases of fatal outcome. 

From March 16, 2020, when the microregion of Tubarão, Santa Catarina, Brazil had the first confirmed case, 210 patients with COVID-19 were referred to the two hospitals that serve the AMUREL region, totaling 30 COVID-19 ICU beds, and wards were provided for the isolation of non-critical patients, with mild to moderate disease symptoms. Compared to the country’s data, there is an increased number of COVID-19 cases and deaths in Santa Catarina after the 27th epidemiological week, which leads to a larger number of hospitalizations and deaths in the surveyed microregion as compared to the whole country^7^. 

Assessment of the cases revealed that older age and the presence of underlying health conditions were risk factors for disease worsening and unfavorable outcomes of SARS-CoV-2 infection. Although in the bivariate analysis, the presence of comorbidities, such as hypertension, heart disease, neurological or psychiatric illnesses, and dyslipidemia were associated with the need for ICU hospitalization, advanced age, obesity, and chronic lung diseases were shown to be independent risk factors for the disease worsening and intensive care unit transfer.

Elderly people are more susceptible to chronic non-communicable diseases and infectious illnesses. Senescence and aging reduce immunity and alter the physiological aspects of several organ systems, thus triggering pathological processes and aggravating the general state of health. As a result, there is an increasing prevalence of polypharmacy, leading elderly people to be more vulnerable to infections, with an ineffective immune response and inadequate antibody production^8^.

During the COVID-19 pandemic, it has been observed that the elderly are more vulnerable to infection with greater disease severity. This fact can be evidenced by the higher number of hospitalizations and deaths among older people. Analyses of age-dependent mortality rates have consistently shown an exponential increase in mortality of COVID-19 patients aged more than 50 years^9^, which was also evidenced in the present study.

Obesity is a condition associated with the worsening of patients infected with SARS-CoV-2. Retrospective cohort studies in Europe and North America have proven the clinical interaction between coronavirus-induced severe acute respiratory syndrome and obesity^10^ ^,^ ^11^. Although it was not initially associated as risk group for COVID-19 infection, obesity is a condition that facilitates the activity of the virus in the patient’s body and can trigger severe clinical conditions^12^.

The immune system of obese patients proves to be dysfunctional in the fight against SARS-CoV-2, from the presentation of the antigen by innate immune cells, when there is a deficit in the activation and availability of cells, mainly macrophages. These cells are constantly activated and concentrated in the adipose tissue and, together with the high load of pro-inflammatory cytokines, cause a series of disorders in tissue homeostasis^12^. 

Another factor triggered by chronic inflammation in obesity is the increase in the number of ACE-2 receptors present in adipocytes. This increase is a consequence of the high concentration of adipocytes and immune cells, which compromises the ACE-2 degradation system. Therefore, the excess of adipose tissue and the increase in the number of receptors for the virus, added to the immune system of the obese, may facilitate viral replication and mutation^13^ ^,^ ^14^.

Moreover, another important factor involved in lung damage is the “cytokine storm”, caused by the high load of pro-inflammatory cytokines present in obesity and the increase in this secretion in the presence of SARS-CoV-2. So far, it is suggested that several unidentified factors may contribute to this phenomenon^12^ ^,^ ^13^.

Although immunological and physiological problems are strongly present in the relationship between obesity and worsening COVID-19, these are not the only ones that promote this association. Other comorbidities associated with obesity, such as the risk of thrombosis caused by fibrinolysis difficulty, vitamin D deficiency, non-alcoholic fatty liver disease (NAFLD), sleep apnea, and intestinal microbiota dysfunction may contribute to the negative evolution of patients infected with SARS-CoV- 2. Unfortunately, not all these parameters were assessed in the sample of the present study. Therefore, further investigation of patients with COVID-19 should be carried out to examine the risk factors or underlying diseases that may interfere with the natural history of COVID-19. All these situations can give rise to complications associated with the severe pulmonary and cardiac impairment observed with this disease^15^ ^,^ ^16^. 

Chronic pulmonary diseases, such as chronic obstructive pulmonary disease, emphysema, and asthma, have also proven to be a risk factor for ICU admission. COVID-19 is primarily a condition of the lower respiratory tract, which may cause viral pneumonia that requires oxygen supply or orotracheal intubation. Chronic pulmonary diseases may alter respiratory physiology, making gas exchange and blood perfusion more difficult^17^.

Critical patients commonly require intensive care and, therefore, a longer hospital stay, which generates a shortage of beds, provoking an overload on the hospital system. 

Among the limitations of this study, it should be mentioned that the research was limited to a single geographical region with few COVID-19 hospital beds, which sometimes required critical patients to be transferred to other regions of the state. With the pandemic still in progress and the acceleration of new cases as of July 2020, some data may change in future analyses, with the inclusion of additional surveyed patients. However, given the scarcity of this type of analysis in Brazil, the local scenario can serve as a comparison to other regions of the country. During the first 120 days of the pandemic, different scientific evidence and isolated experiences indicated the use of some drugs for the clinical management of COVID-19 to avoid complications and deaths resulting from the infection and its complications. Over the period, based on different studies being published, there were changes in the care protocol. In some cases, compassionate drug treatment was provided at the request of the patient or family members. In the present study, some drugs were positively associated with ICU admission and deaths, since the therapy was aimed at severe cases. However, it is noteworthy that the design of this study does not allow for the assessment of pharmacological treatment effectiveness, especially because they are preliminary data covering only four months of patient monitoring. Therefore, the results should be interpreted with caution.

Obesity, chronic pulmonary diseases, and advanced age were independent risk factors for worsening clinical condition requiring ICU admission. Age over 60 years was an independent risk factor for the occurrence of death from COVID-19.

## References

[1] Contini C, Di Nuzzo M, Barp N, Bonazza A, De Giorgio R, Tognon M (2020). The novel zoonotic COVID-19 pandemia: na expected global health concern. J Infect Dev Ctries.

[2] World Health Organization (WHO) (2020). Coronavirus Disease (COVID-19) Pandemic.

[3] Yuki K, Fujiogi M, Koutsogiannaki S (2020). COVID-19 pathophysiology: a review. Clin Immunol.

[4] Siddigi HK, Mehra MR (2020). COVID-19 illness in native and immunosuppressed states: A clinical-therapeutic staging proposal. J Heart Lung Transplant.

[5] Recovery Collaborative Group (2020). Effect of Dexamethasone in Hospitalized Patients with COVID-19 - Preliminary Report.

[6] Atallah B, Mallah SI, AlMahmeed W (2020). Anticoagulation in COVID-19. Eur Heart J.

[7] Ministério da Saúde (MS). Secretaria de Vigilância em Saúde (2020). Doença pelo Coronavírus COVID-19. Semana Epidemiológica 30 (19 a 25 de julho).

[8] Humphreys D, ElGhazaly M, Frisan T (2020). Senescence and Host-Pathogen Interactions. Cells.

[9] Lekamwasam R, Kekamwasam S (2020). Effects of COVID-19 Pandemic on Health and Wellbeing of Older People: a Comprehensive Review. Ann Geriatr Med Res.

[10] Simonnet A, Chetboun M, Poissy J, Raverdy V, Noulette J, Duhamel A (2020). High Prevalence of Obesity in Severe Acute Respiratory Syndrome Coronavirus-2 (SARS-CoV-2) Requiring Invasive Mechanical Ventilation. Obesity.

[11] Kalligeros M, Shehadeh F, Mylona EK, Benitez G, Beckwith CG, Chan PA (2020). Association of Obesity with Disease Severity Among Patients with Coronavirus Disease 2019. Obesity.

[12] Luzi L, Radaelli MG (2020). Influenza and obesity: its odd relationship and the lessons for COVID-19 pandemic. Acta Diabetol.

[13] Ryan PMD, Caplice NM (2020). Is Adipose Tissue a Reservoir for Viral Spread, Immune Activation, and Cytokine Amplification in Coronavirus Disease 2019?. Obesity.

[14] Kruglikov IL, Scherer PE (2020). The Role of Adipocytes and Adipocyte-Like Cells in the Severity of COVID-19 Infections. Obesity.

[15] Muscogiuri G, Pugliese G, Barrea L, Savastano S, Colao A (2020). Commentary: Obesity The “Achilles heel” for COVID-19?. Metabolism.

[16] Zheng Z, Peng F, Xu B, Zhao J, Liu H, Peng J (2020). Risk factors of critical & mortal COVID-19 cases: A systematic literature review and meta-analysis. J Infect.

[17] Pai S, Muruganandah V, Kupz A (2020). What lies beneath the airway mucosal barrier? Throwing the spotlight on antigen-presenting cell function in the lower respiratory tract. Clin Transl Immunology.

